# Implementation of Polygenic Risk Stratification and Genomic Counseling in Colombia: An Embedded Mixed-Methods Study

**DOI:** 10.3390/jpm15080335

**Published:** 2025-08-01

**Authors:** Cesar Augusto Buitrago, Melisa Naranjo Vanegas, Harvy Mauricio Velasco, Danny Styvens Cardona, Juan Pablo Valencia-Arango, Sofia Lorena Franco, Lina María Torres, Johana Cañaveral, Diana Patricia Silgado, Andrea López Cáceres

**Affiliations:** 1 Personalized Medicine Group, Gerencia de Biociencias, Ayudas Diagnósticas Sura, Medellín 050015, Colombia; cbuitragoj@sura.com.co (C.A.B.); hmvelascop@sura.com.co (H.M.V.); dscardona@sura.com.co (D.S.C.); jpvalenciaa@sura.com.co (J.P.V.-A.); sfranco@sura.com.co (S.L.F.); linamariatorressalgado@gmail.com (L.M.T.); ljcanaveralc@sura.com.co (J.C.); cebioentendimiento@suramericana.com.co (A.L.C.); 2 Data Science Department, Gerencia de Biociencias, Ayudas Diagnósticas Sura, Medellín 050015, Colombia; 3 EPS Sura, Medellín 050015, Colombia; dsilgado@sura.com.co

**Keywords:** polygenic risk score, personalized medicine, breast cancer, Latin America

## Abstract

**Background**: Breast cancer remains a major public health challenge in Latin America, where access to personalized risk assessment tools is still limited. This study aimed to evaluate the implementation of a polygenic risk score (PRS)-based stratification model combined with remote genomic counseling in Colombian women with sporadic breast cancer and healthy women. **Methods**: In 2023, an embedded mixed-methods observational study was conducted in Medellín involving 1997 women aged 40–75 years who underwent clinical PRS testing. The intervention integrated PRS-based risk categorization with individualized risk factor assessment and lifestyle recommendations delivered through a remote counseling platform. **Results**: PRS analysis classified 9.7% of women as high risk and 46% as low risk. Healthier lifestyle patterns were significantly associated with lower PRS categories (*p* = 0.034). Physical activity showed a protective effect (OR = 0.60, 95% CI: 0.5–0.8), while prior smoking, elevated BMI, and sedentary behavior were associated with higher risk. The counseling model achieved high delivery (93%) and satisfaction (85%) rates. Qualitative insights revealed improved understanding of genomic risk and greater engagement in preventive behaviors. Only one new case of breast cancer was detected among intermediate-risk participants, with a diagnostic lead time of 12 months. **Conclusions**: These findings support the feasibility, acceptability, and potential impact of integrating PRS and genomic counseling in cancer prevention strategies in middle-income settings.

## 1. Introduction

Breast cancer remains the most frequently diagnosed cancer and the leading cause of cancer-related mortality among women in Latin America, including Colombia [1,2]. Despite advances in early detection and treatment, significant gaps persist in implementing individualized prevention strategies, particularly in low-and middle-income countries (LMICs). Traditional risk assessment approaches—centered on family history or monogenic testing—fail to capture the multifactorial nature of sporadic breast cancer [3,4].

Recent advances in genomic medicine, particularly the development of polygenic risk scores (PRS), have enabled a more precise stratification of risk based on the cumulative effect of multiple common genetic variants [5,6]. When integrated with clinical models and lifestyle factors, PRS can improve risk prediction, support personalized screening strategies, and empower patients to make decisions [7,8]. Studies suggest PRS may identify up to 16% more women eligible for early screening before age 50 [7,9,10]. However, PRS implementation in Latin American populations remains limited, with most models validated primarily in individuals of European ancestry.

In parallel, the field of genetic counseling has evolved. Traditionally focused on monogenic conditions, it has expanded into “genomic counseling” to accommodate more complex genomic data arising from technologies such as next-generation sequencing (NGS) and array-based genotyping [11,12,13]. This transformation has broadened the scope and methodology of counseling, integrated multifactorial risk assessment, and supported behavioral change.

In Colombia, there is no formally established profession of genetic counselors. Instead, around 95 professionals—trained in clinical genetics through a limited number of academic programs—currently deliver genetic counseling services, primarily in urban, private-sector contexts [14]. Additionally, structured models for incorporating PRS into routine preventive care have yet to be evaluated.

In response, Sura Colombia—a healthcare and insurance company—has designed and implemented a two-pronged strategy to enhance risk prediction and prevention for sporadic breast cancer. First, it developed a PRS tailored to the local population to improve risk accuracy. Second, it launched a genomic counseling service to deliver this information through a personalized, clinically integrated model. This study aimed to describe the implementation of a genomic counseling model, focusing on its application to sporadic breast cancer prevention and it evaluates the feasibility, acceptability, and preliminary outcomes of integrating PRS with non-genetic risk factors. The model represents a scalable approach for LMICs seeking to bridge the gap between genomic advances and public health impact.

## 2. Materials and Methods

### 2.1. Setting: “Soy Generación” Study

The “Soy Generación” study was a large observational case–control study conducted in Colombia as part of an initiative to advanced population-specific approaches to breast cancer risk assessment. The study was carried out between July and December 2022 across five major Colombian cities—Bogotá, Medellín, Barranquilla, Bucaramanga, and Cali—and enrolled 1997 women aged 40 to 80 years, including 510 breast cancer cases and 1487 controls. “Soy Generación” focused on evaluating the predictive performance of ancestry-specific polygenic risk scores (PRS) in combination with clinical and imaging risk factors to improve breast cancer risk stratification in Colombian women [15].

Participants were categorized by genetic ancestry—Admixed American, African, and European—and provided detailed clinical data, including breast density and family history. All participants in the main study were offered genomic counseling as part of the return of results process.

In addition, an embedded mixed-methods substudy was conducted on 40 women to describe experiences with the implementation of a genomic counseling model tailored for women with sporadic breast cancer and healthy women in this setting. This component included in-depth interviews and structured surveys to capture perceptions, preferences, and barriers to understanding and acting on genomic information.

#### Variables and Data Collection

Clinical, genetic, and sociodemographic data were systematically collected for all participants through structured interviews, medical records, and molecular assays and using a standardized case report form (CRF). Sociodemographic variables included age, sex assigned at birth, ethnicity, region, place of birth and residence, urban/rural area, and socioeconomic status (classified into six strata). Clinical data comprised reproductive history (age at menarche, first childbirth, and menopause), family history of cancer (in parents, siblings, and aunts), personal history of biopsies, mammographic density (BI-RADS), BRCA1/2 testing results, body mass index at diagnosis and at study entry, and lifestyle factors (smoking, alcohol use, diet, and physical activity). Genetic data encompassed SNP array testing dates, ancestry proportions, and polygenic risk scores (PRS).

### 2.2. Polygenic Risk Score Development and Classification

The polygenic risk score (PRS) used in this study was developed using a Bayesian multivariate regression approach that integrates genome-wide association study (GWAS) summary statistics from multiple ancestries. The model combines data from European, African, and Asian populations and was trained and validated using individual-level data from multiethnic cohorts, including Latin American participants. Genetic ancestry was estimated using 99,561 single nucleotide variants (SNVs) and the iAdmix algorithm with the 1000 Genomes Project reference panel. Principal component analysis was then performed by projecting the individual-level genotype data onto a precomputed 1000 Genomes PCA space, allowing us to estimate ancestry proportions and generate up to 10 principal components for use as covariates.

Samples were genotyped on the Illumina Infinium Global Screening Array v3.0 at the CLIA-certified Omics Science Center at Ayudas Diagnósticas Sura. DNA was extracted from whole blood using the MagMAX DNA Multi-Sample Ultra 2.0 Kit (Thermo Fisher Scientific; Medellín, Colombia) and quantified with Qubit 3.0. Raw genotype data were input to approximately 82 million genome-wide variants using BEAGLE v5.4 and the 1000 Genomes Project v3 reference panel. Quality control and ancestry analyses revealed that over 85% of the cohort had predominantly Amerindian ancestry with varying degrees of European and African admixture.

PRS model construction was performed on the Allelica DISCOVER v1.3 platform based on the PRS-CSx methodology described by Busby et al. (2023) [1,16], selecting SNPs with stringent criteria (*p* < 5 × 10^−8^, LD r^2^ < 0.1, MAF > 1%). The final ancestry-adjusted PRS panel consisted of 313 SNPs. In this Colombian cohort, the model achieved an AUC of 0.72, an OR per SD of 1.56 (95% CI 1.40–1.75), and a Nagelkerke’s R^2^ of 0.14, indicating robust predictive performance across ancestries.

Risk stratification was based on percentiles derived from the distribution of PRS. Four categories were defined: low risk (≤30th percentile), reference (31st–70th), moderate (71st–90th), and high (>90th percentile). Additionally, 10-year absolute risk was estimated using recalibrated risk curves based on local incidence rates, following validated methodologies. All procedures to international PRS reporting standards (PRS-RS), and technical details are available in the Supplementary File S1.

### 2.3. Genomic Counseling Model

**Figure 1:**
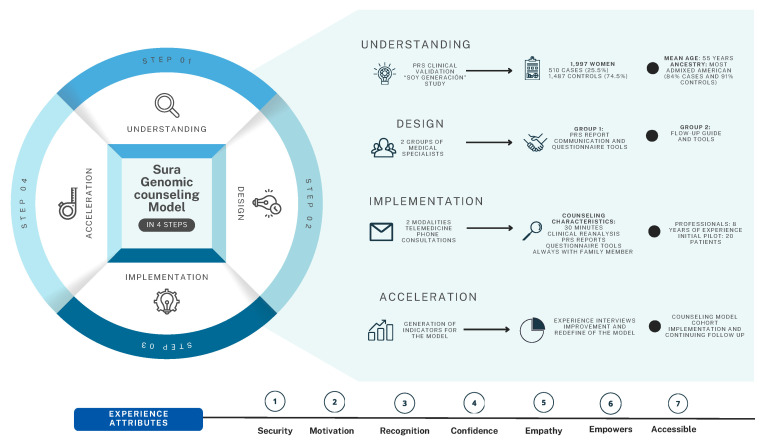
Framework of structured stages of the genomic counseling model in the study.

**Figure 2:**
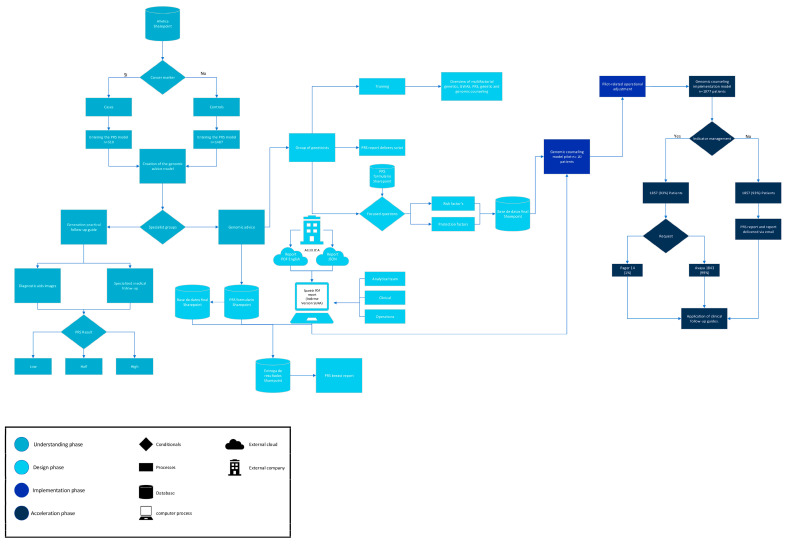
Flowchart of the study design.

The development and implementation of the genomic counseling model followed four structured stages: Understanding, Design, Implementation, and Acceleration (Figure 1). The model was designed in alignment with the Genetic Counseling Intervention Reporting Standards (GCIRS) [17], to ensure transparency, replicability and comprehensive documentation of each component. Details on cohort specification and adherence to GCIRS criteria are provided in Supplementary File S2 and Figure 2.

#### 2.3.1. Understanding Phase

This phase laid the foundation for the genomic counseling model by leveraging the clinical validation findings of the “Soy Generación” study. It included structured training sessions led by experts in genomics, with a particular focus on PRS. This training aimed to strengthen the knowledge base of the clinical team, enabling accurate interpretation and communication of PRS results in the context of breast cancer risk.

#### 2.3.2. Design Phase

During the design phase, two multidisciplinary teams contributed to the development and clinical integration of the genomic counseling model. The first team consisted of clinical geneticists, dysmorphologists, and cancer genetics researchers, with a median of five years of professional experience. This group was responsible for developing three core components of the intervention. First, they designed a standardized PRS report for breast cancer, aligned with both internal quality standards of Sura Colombia and national regulatory requirements. The report provided a clinical interpretation of the PRS, ancestry background information, and personalized lifestyle recommendations tailored to each patient’s genomic profile. Second, the team created a communication script to ensure that results were delivered clearly, consistently, and in a patient-centered manner. This script was intended to support health professionals in translating complex genomic information into language that patients could understand and use. Third, they developed a structured questionnaire to guide the counseling sessions. This tool enabled the collection of relevant clinical and behavioral data, including individual risk and protective factors, indications for physical examination, lifestyle habits, and the patient’s interest in undergoing additional genetic testing.

A second team, composed of breast imaging specialists, mastologists, and oncogeneticists with a median of eight years of clinical experience, contributed by creating a follow-up guide for clinical decision making. This guide was designed to support physicians in managing patients across the spectrum of genomic risk: low, standard, intermediate, and high. The guide was informed by international risk-stratified screening trials such as WISDOM (Women Informed to Screen Depending on Measures of Risk) and MyPeBS (My Personal Breast Screening) [18,19]. Insights from this group were used to refine the genomic counseling model, particularly in terms of translating individual genomic risk into actionable surveillance and clinical pathways.

The entire design phase was supported by an operations team with expertise in innovation, project management, and implementation within health systems. This team developed a digital case report form (CRF) using Microsoft SharePoint, which enables secure and standardized documentation of demographic, clinical, and genomic data across the patient care pathway.

#### 2.3.3. Implementation Phase

The genomic counseling model was piloted using two delivery modalities: telemedicine (video-based sessions) and phone consultations (audio-only). Each session lasted approximately 30 min and was conducted using PAGER or AVAYA platforms. If patients could not be reached, results were shared via email. Counseling sessions included a review of breast cancer risk and protective factors, delivery and explanation of PRS results, and a lifestyle questionnaire addressing diet, alcohol use, and tobacco consumption. All sessions were conducted in the presence of a family member to enhance understanding and support.

The pilot involved five geneticists with an average of 8 years of clinical experience; two held PhDs in human genetics, two had master’s degrees and one had specialized clinical training in genetics. Forty patients participated, recruited from the “Soy Generación” study. Socioeconomic level was determined based on participants’ self-reported housing classification (estrato), which in Colombia reflects a government-assigned socioeconomic stratum ranging from 1 (lowest) to 6 (highest). Rural or urban residence was assessed using each participant’s registered address and categorized according to national criteria.

High-risk individuals were enrolled in a personalized follow-up program and those without a prior cancer diagnosis were offered early detection screening (mammography or MRI, as clinically indicated), while those at low or moderate risk received educational tools focused on prevention and healthy lifestyle promotion. All counseling sessions and reports were provided free of charge as part of the “Soy Generación” study.

This final phase aimed to evaluate and optimize the genomic counseling model prior to broader implementation. Semi-structured interviews were conducted with participants from the pilot study to assess their experiences. The interview guide explored five domains: (1) effectiveness of communication, particularly regarding PRS interpretation; (2) quality of emotional and informational support; (3) relevance and usefulness of lifestyle and clinical recommendations; (4) accessibility and convenience of the counseling modalities; and (5) overall satisfaction with the counseling process.

Findings from the interviews and pilot implementation data informed targeted improvements. These included enhancing the clarity of communication materials, improving patient engagement strategies, and addressing frequently expressed concerns to optimize delivery. Based on these insights, a final version of the counseling model was established.

As part of this refinement, a structured patient journey was developed to guide follow-up care. Patients identified as high-risk were referred to a dedicated clinical pathway for breast cancer surveillance, ensuring continuity of care. Patients with low to moderate risk received tailored educational materials to promote prevention and healthy behaviors. This approach ensured that the genomic counseling model was fully integrated into the broader continuum of care, from risk assessment to preventive intervention.

### 2.4. Clinical Follow-Up and Patient Experience

#### 2.4.1. Clinical Follow-Up

Clinical follow-up was conducted by the research and breast cancer model teams through the “Tiempo para ti” (“Time for You”) program—an initiative designed to provide comprehensive, high-quality care for patients with breast pathology. The program ensured continuous clinical monitoring, personalized risk assessment, and support for patients throughout their care pathways.

Patients identified as low genomic risk were referred to preventive services, including nutrition counseling and health promotion through digital platforms like “Vive más”, which offered personalized programs to improve lifestyle habits [20]. Those with higher risk levels received tailored follow-up based on a structured clinical guide, which included steps from initial screening to long-term surveillance, depending on lesion type and individual risk. A multidisciplinary team coordinated care to ensure timely interventions and standardized practices.

To assess satisfaction and program quality, a structured questionnaire was used during follow-up calls, capturing patient feedback on genomic counseling experience (Supplementary File S4).

#### 2.4.2. Patient Experience

The genomic counseling model was designed with a patient-centered approach, mapping the entire journey from the initial session to clinical or preventive follow-up. This ensured personalized and seamless integration into care pathways.

Educational strategies were central to the experience. Tools like “SENO”, a simple guide for breast self-exams using everyday analogies, encouraged routine self-care (Supplementary File S3) [21]. An educational video explained polygenic risk scores (PRS) and their role in breast cancer prevention in accessible terms, helping patients understand their genetic risk and take proactive steps. A psychologist provided emotional support, helping patients navigate decision making, communicate with family, and manage emotional responses. This was complemented by coaching on healthy behaviors and consistent support from family members.

Feedback mechanisms, such as surveys and interviews, helped improve communication, educational tools, and overall engagement. Digital platforms enhanced access by streamlining scheduling, content delivery, and real-time interaction.

Follow-up and patient experience components combined medical, educational, and emotional support to create a holistic and empowering genomic counseling model for patients.

#### 2.4.3. Statistical Analysis

Descriptive statistics summarized qualitative and quantitative variables. Group comparisons were made using χ^2^, Fisher’s exact, Mann–Whitney, and Kruskal–Wallis tests, with significance set at *p* < 0.05. Normality was assessed using the Shapiro–Wilk test, and odds ratios with confidence intervals were calculated to evaluate associations with lifestyle habits. Polygenic risk stratification was based on prior PRS research. All analyses were performed in R.

For the qualitative component, patient satisfaction data were analyzed using NVivo software version 14, focusing on logistics, infrastructure, doctor–patient interaction, and satisfaction.

## 3. Results

The genomic counseling model was implemented in a cohort of 1997 women, 510 breast cancer cases, and 1487 controls. The median age was 55 years (SD 7) for cases and 70 years (SD 3) for controls. All participants were female, and most lived in urban areas (92% of cases, 93% of controls). Socioeconomic distribution varied: 45% of cases and 33% of controls were in the middle socioeconomic stratum (stratum 3). Significant differences were observed for age at menarche that was slightly lower in cases (median: 12 vs. 13 years, *p* < 0.001). Duration of hormone replacement therapy (HRT) use also differed between groups with a higher proportion of controls having used HRT for more than five years (*p* < 0.0001). Conversely, no statistically significant differences were found for reproductive factors including history of at least one pregnancy, age at first delivery, age at menopause, or the type of hormone replacement therapy regimen.

Polygenic risk score (PRS) stratification showed 44 (8.6%) cases and 144 (9.7%) controls in the high-risk group; 203 (40%) cases and 688 (46%) controls in the low-risk group; 91 (18%) cases and 189 (13%) controls in the moderate-risk group; and 172 (34%) cases and 466 (31%) controls in the reference risk group. Among controls, breast cancer screening methods included mammography (67%), ultrasound (17%), tomosynthesis (15%), and biopsy (0.6%). Sociodemographic variables such as age, residential area, and socioeconomic level showed no statistically significant differences across PRS stratification (Table 1 and Table 2).

In addition, 40 women from the cohort were selected for in-depth interviews and structured surveys to explore their perceptions, preferences, and barriers to understanding and acting on genomic information.

### 3.1. Implementation Outcomes

The genomic counseling model was delivered to all 1997 participants, with 93% (1857) receiving counseling via telehealth or phone sessions and 7% (140) via email. For the qualitative analysis, a purposive subsample of 40 women was selected from the broader “Soy Generación” cohort (*n* = 1997). Among these, satisfaction was high—85% reported being fully satisfied with the counseling process, including result communication and clinical follow-up. Their feedback emphasized themes of personal well-being and family support, as illustrated in the word cloud analysis of qualitative responses, where terms such as “happy”, “family”, “positive”, and “healthy life” were most frequent.

Lifestyle factors significantly influenced breast cancer risk within the cohort. Protective factors, such as physical activity and healthy dietary patterns, were observed in 62% of participants, while 78.1% exhibited two or more risk factors, including sedentary behavior, alcohol consumption, smoking, and diets high in fried or fatty foods. Physical activity was inversely associated with breast cancer risk (OR = 0.6, Cl = 0.5–0.8), whereas prior cigarette smoking was positively associated with breast cancer (OR = 2.031, CI = 1.52–2.71). A greater number of protective factors were identified in controls compared to cases (*p* = 0.0014). Moreover, multiple protective factors in controls correlated with a low-risk PRS stratification (*p* = 0.034). However, no additive association of risk factors with PRS levels was observed in cases (*p* = 0.087) (Table 3 and Table 4).

### 3.2. Clinical Follow-Up and Patient Experience

Mammographic screening was conducted for 965 participants, with 95% classified as low risk (BIRADS categories 1 and 2) and requiring routine population screening. The remaining 5% of participants, categorized as high risk (BIRADS 4 and 5), were incorporated into a diagnostic model involving early detection measures. Among these high-risk participants, one case was diagnosed with early-stage breast cancer and continues to receive appropriate follow-up care. All participants expressed satisfaction with the follow-up process, which was tailored to meet individual risk profiles and needs.

Patient experiences of the genomic counseling model highlighted five core areas: communication effectiveness, patient support, relevance of recommendations, accessibility of counseling, and overall satisfaction. Participants appreciated the clarity in interpreting PRS results and valued the emotional and informational support provided during the counseling sessions. The integration of lifestyle recommendations into the counseling process was well received, with participants acknowledging their relevance and utility in guiding health-related decisions. Telemedicine platforms and phone-based sessions were praised for their accessibility, although areas for improvement were identified such as enhancing follow-up discussions and providing personalized recommendations.

#### 3.2.1. Communication Effectiveness

Participants consistently highlighted the clarity and precision of the explanations provided during the counseling process. Approximately 93.7% of patients stated that the information shared about polygenic risk scores (PRS), genetic testing procedures, and their implications was thorough and easy to comprehend. The role of the genetics specialist in simplifying complex genomic concepts was particularly appreciated, as it helped alleviate initial concerns about the testing process and results interpretation. Participants shared deeply personal reflections that highlighted the transformative impact of genomic counseling on their perspectives and behaviors. Many emphasized how the process motivated them to reassess and prioritize their health. One participant remarked

*“I have two challenges: letting go of paradigms and many thoughts. When I bring an important piece of genetic health information from my parents, it acts as a warning signal—something to pay attention to. It’s a chance to prioritize which health conditions I need to address first. Being part of this process, which has the potential to change the lives of many women, feels like a beautiful gift. It’s helped me change my physical activity routine. While at times it brings anxiety and less pleasant emotions, it also offers the opportunity to think differently, to face fears and anxieties, and to see these as part of the process.”*

Participant 20

Another participant reflected on how the model brought both personal and familial clarity:

*“It’s been excellent—very good and reassuring. For context, someone in my family passed away from breast cancer that metastasized. We lived through the entire process without any improvement, and it left a permanent mark on me. When I was called to participate, I felt a little scared and thought, ‘Oh my God, could something bad happen?’ But everything turned out to be a blessing. Being part of this model has made me more conscious. It became a personal challenge to make changes. With the ‘Vive Más’ platform, I started moving more, dancing, and seeking change. Today, I feel at peace after this test.”*

Participant 2

These statements reflect the emotional journey that participants underwent during genomic counseling. Patients expressed a balance between initial anxiety and ultimate reassurance, as well as a profound appreciation for the opportunity to gain insights into their genetic risks. The clarity of communication allowed participants to better understand the implications of the information provided, fostering empowerment and a sense of personal agency in addressing their health.

#### 3.2.2. Patient Support

Patients expressed profound appreciation for the emotional and informational support provided through the counseling model. Key themes emerged from their feedback, highlighting gratitude, reassurance, and a sense of empowerment derived from their participation. One participant shared their initial apprehension, which transformed into gratitude after understanding the purpose of the model:

*“When I was invited to be part of the model, I thought, ‘Why me? Did they find something?’ But when they explained it to me, I realized what a gift the company was giving us.”*

Participant 30

Clarity in communication and the professionalism of the healthcare team were recurring points of praise. A participant highlighted

*“The clarity of the explanations was remarkable. They told us, ‘This process is to identify the probability of risk.’ The specialized exams and the warmth of everyone involved in the process made me feel valued. On the day of the tests, I felt like a queen—it has such an integral approach.”*

Participant 9

Many patients reported a sense of tranquility during and after the counseling sessions. One stated

*“I feel reassured, knowing this is about prevention and care. The genetic percentage and the sporadic percentage were explained so clearly. I feel accompanied by experts who guide me and show progress every step of the way.”*

Participant 5

The comprehensive support offered by the genomic counseling team fostered trust and confidence. Participants appreciated having access to allied health professionals to address their questions and guide them through logical steps, which many described as seamless and thoughtful. This combination of empathy, clarity, and professionalism enhanced patients’ perception of the genomic counseling model, making it not only an educational experience but also an emotionally supportive one.

#### 3.2.3. Relevance of Recommendations

**Figure 3:**
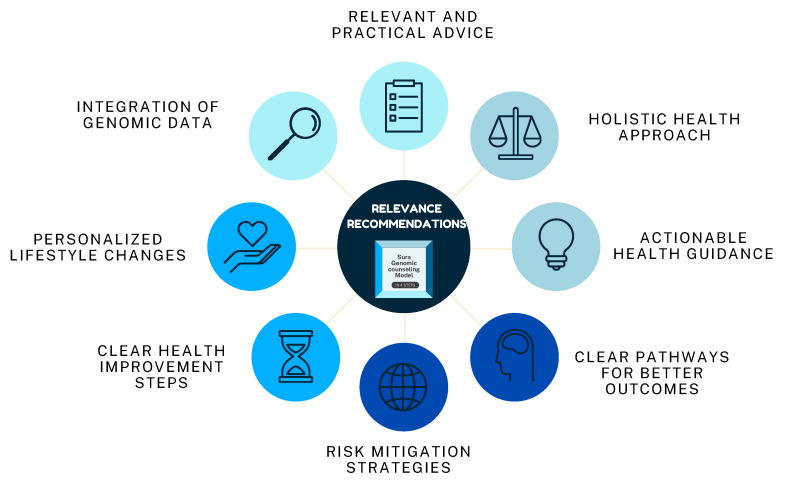
Feedback loop. The genomic counseling model was highly praised for its integration of personalized lifestyle recommendations, which participants found relevant, actionable, and effective in mitigating breast cancer risk. Patients appreciated the clear guidance provided, emphasizing the practical combination of genomic and non-genomic insights to improve health outcomes. However, areas for improvement include expanding accessibility, ensuring culturally sensitive communication, and providing follow-up support to enhance the long-term impact of the recommendations.

One of the standout aspects of the genomic counseling model was the integration of tailored lifestyle recommendations into the counseling sessions. Participants reported finding this suggestion highly relevant and actionable, particularly in the context of mitigating breast cancer risk. Many patients expressed that the recommendations provided a clear pathway to improve their health outcomes, emphasizing the practical utility of combining genomic data with non-genomic insights. Key highlights are shown in Figure 3.

#### 3.2.4. Accessibility of Counseling

The use of telemedicine platforms and phone-based consultations greatly improved the accessibility of genomic counseling, making it easier for patients to engage in sessions without logistical challenges. Many participants appreciated the convenience of remote interactions, particularly for reducing travel time and costs. One patient shared that “Being able to join from home made it much easier with my busy schedule”. This was especially beneficial for those in rural or underserved areas who otherwise would have faced difficulties accessing care.

However, some patients noted that phone consultations felt less personal. One participant mentioned

*“I felt the phone session was helpful, but a video call would have felt more connected”*

Participant 10

Suggestions for improvement included more video interactions and ensuring thorough follow-up discussions to address lingering concerns. Overall, the accessibility of counseling was positively received, with patients appreciating the convenience and flexibility of remote options. As one participant summarized,

*“Telemedicine made it easy to focus on my health without worrying about logistics”.*

Participant 3

#### 3.2.5. Overall Satisfaction

The overall patient satisfaction with the genomic counseling model was high, with 96% of participants reporting a positive experience. Key factors contributing to this satisfaction included the clarity of communication, the personalized attention provided, and the efficient coordination of services. Participants consistently described the process as well organized, with one stating

*“The whole process was seamless; from the moment I was invited to join until the final step. It felt very well-coordinated”.*

Participant 18

The emotional and informational support provided throughout the counseling session was frequently highlighted. Many participants described the experiences as “supportive”, “informative”, and “empowering”, with several noting that the counseling helped them feel more confident in making informed decisions about their health. One participant remarked

*“I felt like I was really part of the process and that I had all the information I needed to take care of myself”.*

Participant 22

Moreover, the integration of both genetic and lifestyle factors in the counseling process was highly valued. Participants emphasized that understanding these factors provided them with a sense of control over their health. As one patient noted,

*“It gave me the tools to take proactive steps, knowing both my genetic risk and what I can do about it”.*

Participant 40

These findings underscore the effectiveness of the genomic counseling model in promoting informed decision making and providing patients with a heightened sense of control over their health outcomes.

## 4. Discussion

The implementation of genomic counseling for breast cancer patients in Colombia, particularly for those with polygenic risk scores (PRS), presents a series of complex challenges. The primary difficulty stems from the absence of clinical validation studies in Colombia, with cross-comparison data being limited. Notably, clinical practice guidelines supporting the integration of genomic data into routine practice are not yet established within the country. This absence of evidence base and supportive guidelines has created barriers in the implementation of our model [22]. In addition to these scientific and infrastructural challenges, cultural barriers within the medical community and among patients have posed significant obstacles to effective implementation. Our study represents the first attempt to integrate genomic counseling within a personalized primary prevention model for sporadic breast cancer in Latin America [14].

A critical issue identified during our genomic counseling implementation was the lack of clinical validation studies specific to the Colombian population. Our efforts to generate such data through a PRS validation process were essential, given that no prior studies have been conducted on the Colombian population to facilitate cross-comparison. Furthermore, there is no formal curriculum in Colombian medical training programs that includes genomic counseling as part of the genetics specialty, which creates a gap in the availability of qualified healthcare professionals to conduct such counseling [23]. The complexity of explaining the risk of multifactorial diseases like breast cancer using genomic information such as common variants, particularly PRS, added to the difficulty in communicating such complex data effectively to patients [22,24,25].

To address the existing barriers, a two-stage personalized prevention approach was implemented. The first stage focused on primary prevention, recommending protective measures and intervention for modifiable risk factors, while the second stage aimed at secondary prevention through early cancer detection using advanced diagnostic technologies [10,24]. The approach also included the clinical validation of PRS for breast cancer, given the lack of population-specific data in Colombia. Our risk stratification (low risk: 46%, baseline: 31%, moderate: 13%, high: 9.7%) aligns with the expected distribution in the general population. Clinical studies on PRS in European and Hispanic populations show similar findings, with odds ratios (OR) of 1.55 and an area under the curve (AUC) of 0.79, reinforcing the validity of PRS as a tool for risk assessment in Latin American populations, despite the lack of local validation [24,26,27].

Incorporating the European Code Against Cancer’s recommendations into our intervention enhances its impact. These guidelines emphasize lifestyle changes—avoiding tobacco, maintaining a healthy weight, engaging in physical activity, and consuming a balanced diet rich in fruits and vegetables. They also highlight the importance of early cancer detection through screening and vaccination programs, such as HPV vaccines. By integrating these preventive strategies with genomic counseling, our approach offers a comprehensive, evidence-based framework to reduce cancer risk and promote early detection in the Colombian population [4,7].

In our study, we implemented a follow-up methodology that included mammography, ultrasound, and breast MRI based on individual risk stratified by PRS for 965 women without a breast cancer history. Notably, only one patient in the intermediate-risk group was diagnosed with in situ breast cancer, underscoring the value of early detection. Although our methodologies align with ongoing randomized clinical trials, such as WISDOM and MyPeBS, differences in risk stratification, ethnic composition, and geographical context highlight the importance of locally adapted screening protocols tailored to the population’s characteristics for optimal breast cancer prevention [18,19,28]. Additionally, our study identified key lifestyle factors influencing breast cancer risk. Smoking significantly increased the risk (OR = 2.031, CI = 1.52–2.71), while physical activity acted as a protective factor (OR = 0.6, CI = 0.5–0.8), aligning with findings from the UK BioBank and other cohort studies. These results emphasize the importance of combining healthy lifestyle promotion with genomic risk data to reduce breast cancer incidence effectively [29,30,31].

Finally, the implementation of genomic counseling for breast cancer in Colombia, particularly for PRS patients, represents a significant step forward in personalized medicine. While there are considerable challenges, the structured approach of technical validation, education, and guideline development provides a robust framework for integrating genomic data into clinical practice [32]. Continued efforts in research, education, and patient engagement will be essential to fully realize the benefits of this innovative approach.

## 5. Conclusions

This study demonstrates the feasibility and potential impact of implementing a PRS-based risk stratification model combined with remote genomic counseling in both women with sporadic breast cancer and healthy women. By integrating genomic information into clinical care pathways, this strategy enables earlier and more accurate risk assessment, promotes personalized plans, and supports timely detection of new cases.

For healthy women, PRS allows the identification of those at elevated risk who may benefit from targeted prevention and lifestyle interventions. For women with a history of breast cancer, the model facilitates tailored follow-up and supports the implementation of precision strategies aimed at secondary prevention.

Empowering women with genetic risk information fosters engagement, improves shared decision making, and can ultimately reduce morbidity and mortality through timely interventions. This model, if scaled and adapted, could serve as a blueprint for integrating precision prevention into cancer control strategies in other middle-income settings.

## Figures and Tables

**Table 1:** Sociodemographic characterization of the patients in the study.

Characteristics	Control, N = 1487 ^1^	Case, N = 510 ^1^
Age	69.7 (67–71)	55 (49–61)
Rural or urban residence		
Rural	107 (7.2%)	39 (7.6%)
Urban	1380 (93%)	471 (92%)
Socioeconomic level		
1	53 (3.6%)	29 (5.7%)
2	269 (18%)	138 (27%)
3	490 (33%)	232 (45%)
5	357 (24%)	71 (14%)
5	233 (16%)	29 (5.7%)
6	85 (5.7%)	11 (2.2%)
Clinical risk classification		
High (>1.9)	144 (9.7%)	44 (8.6%)
Low (<1)	688 (46%)	203 (40%)
Moderate (1.4–1.9)	189 (13%)	91 (18%)
Referent (1–1.4)	466 (31%)	172 (34%)
Breast cancer diagnosis		
Biopsy	3 (0.6%)	-
Breast echography	80 (17%)	-
Mammography	315 (67%)	-
Tomosynthesis mammography	73 (15%)	-

^1^ n (%).

**Table 2:** “Soy Generación” study population characteristics about clinical validation in control group.

Characteristics	High (1.9), N = 144 ^1^	Low (<1) N = 688 ^1^	Moderate (1.4–1.9) N = 189 ^1^	Referent (1–1.4) N = 466 ^1^	*p*-Value ^2^
Age	69.9 (3.4)	69.7 (3.5)	69.4 (3.3)	69.8 (3.6)	0.4
Rural or Urban residence					0.8
Rural	8 (5.6%)	53 (7.7%)	15 (7.9%)	31 (6.7%)	
Urban	136 (94%)	635 (92%)	174 (92%)	435 (93%)	
Socioeconomic level					0.9
1	3 (2.1%)	26 (3.8%)	5 (2.6%)	19 (4.1%)	
2	23 (16%)	125 (18%)	35 (19%)	86 (18%)	
3	48 (33%)	228 (33%)	60 (32%)	154 (33%)	
4	33 (23%)	157 (23%)	56 (30%)	111 (24%)	
5	29 (20%)	110 (16%)	25 (13%)	69 (15%)	
6	8 (5.6%)	42 (6.1%)	8 (4.2%)	27 (5.8%)	

^1^ n (%), Mean (SD), ^2^ Kruskal-Wallis rank sum test; Pearson’s Chi-squared test.

**Table 3:** Protective and risk factors of patients in the study.

Variable	Case, N = 457 ^1^	Control, N = 1283 ^1^	*p*-Value ^2^
Protective factors			
Diet *	431 (94%)	1188 (93%)	0.2
Physical activity **	257 (56%)	900 (70%)	<0.001
Risk factors			
Diet with fats	328 (72%)	904 (70%)	0.6
Active smoking	11 (2.4%)	44 (3.4%)	0.3
Former smoker	58 (13%)	318 (26%)	<0.001
Alcohol	76 (17%)	171 (13%)	0.082

^1^ n (%). ^2^ Pearson’s Chi-squared test. * Diet was defined as adherence to “frequent intake of fruits, vegetables and low-fat foods”. ** Physical activity was defined as ≥150 min of moderate-to-vigorous exercise per week.

**Table 4:** Risk estimation by lifestyle in the study.

Variable	OR	*p*-Value	IC
Protective factors			
Diet	1.326	0.217	(0.86, 2.113)
Physical Activity	0.547	0.000	(0.439, 0.682)
Risk Factors			
Diet with Fats	1.066	0.596	(0.843, 1.353)
Active Smoking	0.695	0.286	(0.338, 1.308)
Former smoker	0.435	0.000	(0.318, 0.585)
Alcohol	1.297	0.083	(0.963, 1.735)

## Data Availability

The datasets used and/or analyzed during the current study are available from the corresponding author on reasonable request.

## Supplementary Materials

The following supporting information can be downloaded at: https://www.mdpi.com/article/10.3390/jpm15080335/s1, Supplementary File S1: Risk stratification methodology and PRS origin and classification; Supplementary File S2: Checklist for reporting genetic counseling interventions applied in our study; Supplementary File S3: “SENO” (Which means “breast” in Spanish) tool, an innovative educational tool designed to teach women how to perform self-breast exams effectively by Sura Colombia; Supplementary File S4: Patient Satisfaction Questionnaire for the “Linea rosa” in the breast cancer model.

## Abbreviations

PRS	Polygenic risk score
LMICs	Low-and middle-income countries
NGS	Next-generation sequencing
GCIRS	Genetic Counseling Intervention Reporting Standards.
WISDOM	Women Informed to Screen Depending on Measures of Risk
MyPeBS	My Personal Breast Screening
CRF	Digital Case Report form
BIRADS	Breast Imaging Reporting and Data System
