# Supplementary material for: Implementation of Polygenic Risk Stratification and Genomic Counseling in Colombia: An Embedded Mixed-Methods Study
Source: J Pers Med. 2025 Aug 1;15(8):335. doi: 10.3390/jpm15080335 (PMC12387499; doi:10.3390/jpm15080335)
Supplement: Supplementary file 1 [file jpm-15-00335-s001.zip › jpm-3698859-supplementary.pdf]

Supplementary Appendix 1:

Risk Stratification Methodology

Polygenic Risk Scores (PRS) were calculated using a proprietary model provided by Allelica Inc., based on a panel of common single nucleotide polymorphisms (SNPs) previously validated in studies of breast cancer susceptibility. Scores were calculated for each participant using a weighted sum of risk alleles. To enable stratification, the distribution of PRS in the control group (n = 1,487) was used to define risk thresholds.

Participants were categorized into four strata according to the following PRS percentile-based cutoffs:
- Low risk: Below the 30th percentile
- Reference risk: 30th to 70th percentile
- Moderate risk: 71st to 90th percentile
- High risk: Above the 90th percentile

These thresholds align with prior literature including Shieh et al. (2020), and allow for meaningful separation of predicted risk.

Clinical characteristics, sociodemographic data, and lifestyle variables were cross-tabulated by PRS category and assessed using chi-square and Kruskal-Wallis tests. Results from this stratification are summarized in Tables 1 and 2 of the manuscript.

PRS Origin and Classification Appendix

The PRS used in this study was developed and provided by Allelica Inc. and constructed using the Allelica DISCOVER v1.3 platform, which implements the PRS-CSx methodology described by Busby et al. (2023). Single nucleotide polymorphisms (SNPs) were selected under stringent criteria ($p < 5\times10^{-8}$, LD $r^2 < 0.1$, MAF > 1%) from genome-wide association study (GWAS) summary statistics of European, African, and Asian ancestry populations. The final ancestry-adjusted PRS panel consisted of 313 SNPs.

The model was externally validated in this Latin American cohort of 1,997 Colombian women, who were predominantly of admixed American ancestry (>85%). In this population, the PRS achieved an area under the curve (AUC) of 0.72, an odds ratio per standard deviation of 1.56 (95% CI 1.40–1.75), and a Nagelkerke's R^2 of 0.14, demonstrating robust predictive performance.

As previously described in the main manuscript, the cohort was stratified into four risk categories (low, reference, moderate, and high) using control group percentiles as cutoffs. Results from this stratification are shown in Tables 1 and 2 of the main text.

Reference.

Shieh Y, Hu D, Ma L, Huntsman S, Gard CC, Leung JWT, et al. Breast cancer risk prediction using a clinical risk model and polygenic risk score. Breast Cancer Res Treat. 2016 Oct 1;159(3):513–25.

Busby, G.B., Kulm, S., Bolli, A. et al. Ancestry-specific polygenic risk scores are risk enhancers for clinical cardiovascular disease assessments. Nat Commun 14, 7105 (2023). https://doi.org/10.1038/s41467-023-42897-w

Supplementary appendix 2. Checklist for reporting genetic counseling interventions applied in our study.

Component	Description	Meets criteria	Don´t meet criteria
**1. Indication for genetic counseling**			
1.1 Reason for genetic counseling intervention	In the introduction and methods of the study specified. Our objective with the genetic counseling model was in part to lead the development of regional redefinitions for conventional risk approaches in sporadic breast cancer implementing PRS (Lines 80-89).	X	
1.2 Affected status of the counselee (clinically symptomatic/asymptomatic) at time of genetic counseling intervention.	In the methods we specified that the inclusion of patients was intended to assess effectiveness of the genetic counseling model across different risk categories, refine the approach based on their specific needs and to better integrate genetic counseling into routine clinical practice for breast cancer risk management (Lines 118-124)	X	
**2. Other Components of a Complex Intervention**			
2.1 Other evaluations at the time of genetic counseling interventions (e.g., other clinician's interactions, physical examination)	The implementation of the genomic counseling model was through two pathways: 1) Counseling via telemedicine and 2) Phone Call. There were no other evaluations during the genetic counseling intervention (Lines 154-175).	X	
2.2 Genetic testing before, after, or at the time of counseling	Patients were part of an observational case-control study which aimed to validate a polygenic risk score for breast cancer tool.	X	
2.3 Testing indications or risk thresholds for testing	Not applied. Patients were part of a case-control study which aimed to validate a polygenic risk score for breast cancer tool (no specific indication for the study).	X	
**3. Intervention Delivery**			
3.1 Delivery mode (telephone, in person, telemedicine, video; with interpreter; individual, couple or group; if group, group size and composition).	The delivery of the genomic counseling was via telemedicine, 30 minutes per patient (Lines 149-161).	X	
3.2 Physical setting (hospital, clinic, public vs. private)	Patients were affiliates through Sura Colombia National program (a health insurance company in Colombia). The genomic counseling model was implemented through telemedicine with genomics experts that works at the Sura Omics Science Center.	X	
3.3 Payment method (genetic counseling paid for by participant, private or government insurance, or grant funding, whether participants received incentive)	The delivery of the results and the genetic counseling provided to the patients was part of the experience build in "SOY GENERACIÓN" project and patients did not have to pay for the service (Lines 160-163).	X	
**4. Provider(s) of Genetic Counseling**			
4.1 Qualifications or other training/credentials	Genomic counseling model was executed by geneticists (5 in total) with 2 held PhDs in human genetics, 2 had master´s degrees and 1 had a specialization in genetics, with an average of 8 years of experience.	X	
4.2 Number and type(s) of healthcare professionals involved in the genetic counseling interaction with each participant	Each interaction was made with 1 healthcare professional (Geneticists).	X	
4.3 Number and type(s) of healthcare professionals delivering intervention in study	Genetic counseling was delivered by Geneticists. But all the model was supported by healthcare professionals that included: physicians, nurses and an operational team.	X	
**5. Risk Content and Communication**			

5.1 Basis of risk assessment (family history, test results, personal history tools and algorithms)	Risk assessment involved: family history, test results (PRS), personal history tools and algorithms.	**X**	
5.2 Type and format of risk information provided to participant (e.g., frequencies, odds ratios, absolute/relative risk etc.)	Polygenic Risk Scores (PRS) for breast cancer are a tool used to estimate an individual's genetic predisposition to developing the disease. PRS are calculated by analyzing multiple genetic variants across the genome, each contributing a small amount to the overall risk. By combining these variants, a cumulative risk score is generated, helping to identify individuals at higher or lower risk for breast cancer. This personalized risk assessment can be used to guide prevention strategies, screening recommendations, and early detection efforts.	**X**	
**6. Educational Content**			
6.1 Educational goals and learning objectives of genetic counseling intervention.	Educational content was construct during all the implementation models. Please see lines 191-197.	**X**	
6.2 Educational tools employed (visual aids, interactive web applications, others)	Interactive web applications, apps, visual aids for patients and videos where used.	**X**	
6.3 Educational models or theories applied	Two educational models where used: Health Belief Model (HBM) and Transtheoretical Model (Stages of Change) (Lines 205-212).<br>**Health Belief Model (HBM):** This model is effective because it focuses on individual perceptions of risk, which is crucial when dealing with Polygenic Risk Scores (PRS). Patients may be more inclined to adopt preventive measures, such as lifestyle changes or additional screenings, if they understand their genetic susceptibility to breast cancer and the benefits of early intervention. HBM helps address perceived barriers to taking preventive actions, such as fears about the efficacy of interventions or lack of understanding about PRS. It facilitates informed decision-making regarding health actions based on genetic risk.<br>**Transtheoretical Model:** In the context of PRS for breast cancer, this model allows genetic counselors to identify where patients stand in terms of readiness to make lifestyle changes or undergo additional screenings. Since PRS provides information about the risk of developing breast cancer, it's essential for patients to be ready to act on this information. This model is useful for guiding patients through the stages of preparation, action, and maintenance of healthy behaviors. It's key to ensure that high-risk patients implement preventive changes, such as increased monitoring or changes in diet and exercise.	**X**	
**7. Psychotherapeutic Content**			
7.1 Psychotherapeutic goals (e.g., decision making, promoting family communication, facilitating coping and adaptation)	Psychotherapeutic content was effectively integrated to support decision-making, enhance family communication, and facilitate emotional coping and adaptation. Emotional monitoring associated with participation was conducted by a psychologist, who utilized various communication channels across different locations, ensuring thorough follow-up on requests, complaints and feedback.	**X**	
7.2 Psychotherapeutic tools and techniques employed.	Techniques such as coaching on healthy models and mental health were implemented, with patients	**X**	

	consistently accompanied by a family member. This approach validated their understanding and reinforced the sense of support, ensuring that patients felt accompanied throughout the process.		
7.3 Psychotherapeutic models or theories.		**X**	
**8. Duration**			
8.1 Length of each genetic counseling interaction.	30 minutes for consultation per patient	**X**	
8.2 Number of genetic counseling interactions.		**X**	
8.3 Time between genetic counseling interactions.	2 minutes per patient.	**X**	
8.4 Follow-up genetic counseling interactions	All patients who had high-risk were included in “TIEMPO PARA TI” program. Patients with low to moderate risk were provided with educational tools for breast cancer prevention and promotion, developed by Sura Colombia breast cancer model.	**X**	

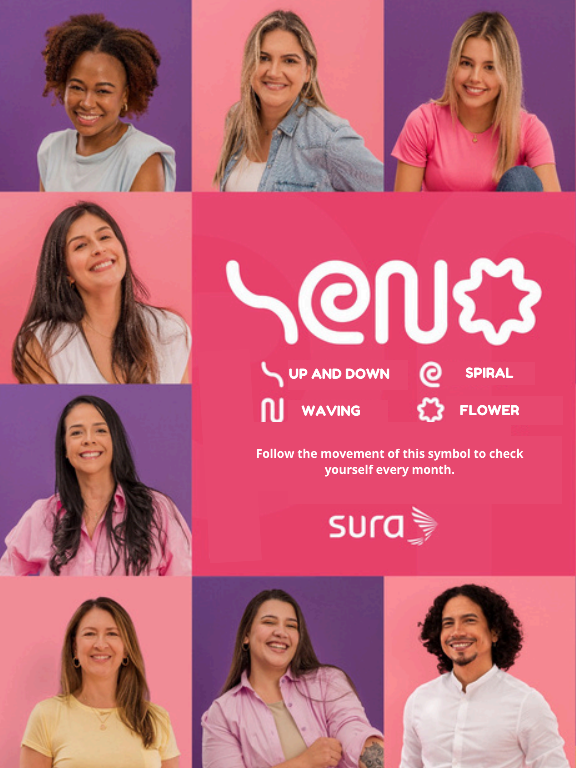

UP AND DOWN

Using three fingers, feel the entire surface of your breast from the armpit to the lower part

SPIRAL

Place two fingers in the center of the nipple and start sliding them in a spiral motion until you reach the edge of the breast.the movement

WAVING

Move the middle fingers in a vertical line, from the top to the bottom of the breast, and advance in a wavy motion across the entire breast.

FLOWER

Gently squeeze your nipple to check for any lumps or fluid secretion.

Supplementary Appendix 4: Patient Satisfaction Questionnaire for the "LINEA ROSA" in the breast cancer model.

To further improve patient care and experience, a satisfaction questionnaire tool was implemented as part of the breast cancer program follow-up calls. This tool assessed patient satisfaction with the breast cancer program and the genomic counseling model´s implementation.

The "Linea Rosa – Pink line" is a dedicated service providing support to individuals seeking guidance on the breast cancer model. Through this service, users can access support via customer service at **01 8000 519 519** or via WhatsApp. Patients can also request medical appointments through their assigned family physician or via website. As part of the genomic counseling model, this service was integrated to monitor and enhance patient satisfaction.

Patient Satisfaction Questionnaire

1. How satisfied or dissatisfied were you with the care received? – Select one option:
    a. 1: Totally dissatisfied.
    b. 2: Dissatisfied
    c. 3: Neither satisfied nor dissatisfied
    d. 4: Satisfied
    e. 5: Totally satisfied
2. Did the options available through the "Linea Rosa – Pink line" resolve your needs related to breast pathology?
    a. 1: Yes
    b. 2: No
3. How likely are you to recommend the "Linea Rosa – Pink line" service to your family, friends, colleagues, or acquaintances? – Rate and select one option, being 0: Extremely unlikely and 10: Extremely likely

This tool enabled the team to evaluate the program´s effectiveness and identify opportunities for improvement to ensure optimal patient-centered care.
